# Adaptive Multi-Type Fingerprint Indoor Positioning and Localization Method Based on Multi-Task Learning and Weight Coefficients *K*-Nearest Neighbor

**DOI:** 10.3390/s20185416

**Published:** 2020-09-21

**Authors:** Zhengwu Yuan, Xupeng Zha, Xiaojian Zhang

**Affiliations:** School of Computer Science and Technology, Chongqing University of Posts and Telecommunications, Chongqing 400065, China; yuanzw@cqupt.edu.cn (Z.Y.); 18237508539@163.com (X.Z.)

**Keywords:** multi-type fingerprints, indoor positioning and localization, multi-task learning, weight coefficients *K*-Nearest neighbor

## Abstract

The complex indoor environment makes the use of received fingerprints unreliable as an indoor positioning and localization method based on fingerprint data. This paper proposes an adaptive multi-type fingerprint indoor positioning and localization method based on multi-task learning (MTL) and Weight Coefficients *K*-Nearest Neighbor (WCKNN), which integrates magnetic field, Wi-Fi and Bluetooth fingerprints for positioning and localization. The MTL fuses the features of different types of fingerprints to search the potential relationship between them. It also exploits the synergy between the tasks, which can boost up positioning and localization performance. Then the WCKNN predicts another position of the fingerprints in a certain class determined by the obtained location. The final position is obtained by fusing the predicted positions using a weighted average method whose weights are the positioning errors provided by positioning error prediction models. Experimental results indicated that the proposed method achieved 98.58% accuracy in classifying locations with a mean positioning error of 1.95 m.

## 1. Introduction

With the speedy development of mobile communication technology and the Internet of Things, indoor positioning and localization technology have attracted widespread attention as an essential part of location-based services. In recent years, researchers have proposed some indoor positioning and localization methods based on different technologies, such as Ultra-wideband [1,2] (UWB), Bluetooth [3,4,5], Wireless Fidelity [6,7,8] (Wi-Fi), Radio Frequency IDentification [9,10] (RFID), and magnetic field [11,12,13] (MF). Among them, the MF, Wi-Fi, and Bluetooth technologies have some advantages (such as easy deployment, low cost), which provide many applications in diverse fields, such as asset tracking, patient monitoring, warehouse management, and crowd analysis, etc. With the rapid popularization and application of mobile terminals, receiving MF, Wi-Fi, and Bluetooth signals become more real-time and convenient. Thus, with the popularization and application of MF, Bluetooth, and Wi-Fi methods based on these three technologies will likely become ordinary for indoor positioning and localization.

Fingerprint positioning and localization have the advantages of simple construction, low cost, easy implementation, and low maintenance, which is a universal method for positioning and localization based on MF, Wi-Fi, and Bluetooth. Today, there have been many studies on MF fingerprint [14,15] positioning and localization [16,17], Wi-Fi fingerprint positioning and localization [18,19], as well as Bluetooth fingerprint positioning and localization [20,21], but studies on the integration [22,23] of MF, Wi-Fi and Bluetooth fingerprints for positioning and localization have been comparatively few. Nevertheless, all the MF, Wi-Fi, and Bluetooth signals are present in certain indoor environments, such as office buildings, supermarkets, warehouses. Therefore, it is necessary to study the integration of MF, Wi-Fi, and Bluetooth fingerprints to make full use of existing signals for indoor positioning and localization.

A few works have investigated fusion positioning and localization using two fingerprints in MF, Wi-Fi, and Bluetooth fingerprints. In the work of [24], an F-score-weighted indoor positioning algorithm was proposed, which integrated MF and Wi-Fi fingerprints. In [25], Baniukevic et al. introduced Bluetooth hotspots into an indoor place with an existing Wi-Fi infrastructure, which is better than positioning using each technology alone. An adaptive Bluetooth/Wi-Fi fingerprint positioning method based on Gaussian process regression and relative distance was proposed in [26], which can choose a more reliable positioning result as the final positioning result. The experimental results of the above studies demonstrate that the fusion positioning and localization method can achieve better positioning and localization performance compared to using a single type of signal. Nevertheless, most of the fusion positioning and localization methods still have some challenges. For instance, the potential relationship between different types of fingerprints is not considered, which leads to the loss of some information. Moreover, most of the positioning and localization algorithms are not suitable for the environment of multi-type fingerprints, thus the positioning and localization effects need to be further improved.

This paper proposes an adaptive multi-type fingerprints indoor positioning and localization method based on multi-task learning (MTL) and Weight coefficients *K*-Nearest Neighbor (WCKNN). The method is principally divided into two stages: training and testing. In the training stage, we develop an MTL [27,28] model to simultaneously predict the symbol location and position coordinate of multi-type fingerprints and refine the weight coefficients of WCKNN using the relationship between the attributes of the training data. Then, positioning error prediction models based on Deep Neural Networks [29,30] (DNN) are established and trained using processed validation data. In the testing stage, the MF, Wi-Fi, and Bluetooth fingerprints of test data are input into the MTL model to get the symbol location and position coordinate of the fingerprints. Subsequently, another position coordinate is obtained by using the WCKNN in the clusters determined by the obtained symbol location. The clusters are composed of fingerprints with the same symbol location in the training and validation datasets and can reduce the positioning range of WCKNN. And so, with the help of positioning error prediction models, the positioning error of each predicted position coordinate can be determined, which represents the confidence of the position coordinate. Finally, the final position coordinate is obtained by fusing the two predicted position coordinates using a weighted average method whose weights were the positioning errors. The second section of the paper introduces the dataset adopted and the algorithms involved in this study. The third section presents the proposed method in detail. The fourth section introduces the results of the study and compares them with current results. Finally, some conclusions are presented based on the results. Moreover, there are some contributions to this paper as follows.

- The designed MTL model fuses MF, Wi-Fi, and Bluetooth fingerprint features by a hard parameter sharing mechanism to predict the position and the location of the fingerprints, which can learn the potential relationship between fingerprints and improve the performance of positioning and localization to a certain extent.
- A *K*-Nearest Neighbor [31,32] (KNN) algorithm based on weight coefficients is proposed, which carries the influence of different types of fingerprints on the similarity between multi-type fingerprints into consideration.
- The positioning error prediction model can estimate the confidence of the position coordinate to realize adaptive indoor positioning.

## 2. Materials and Methods

According to the needs of users, fingerprint indoor positioning and localization are usually split into two types of tasks: regression and classification [33]. The regression task is to predict the position information of fingerprint, corresponding to a specific point in a coordinate system such as longitude and latitude. In contrast, the classification task is to predict the location information of the fingerprint, which can provide detailed information for the position, such as office, floor, building.

**Figure 1:**
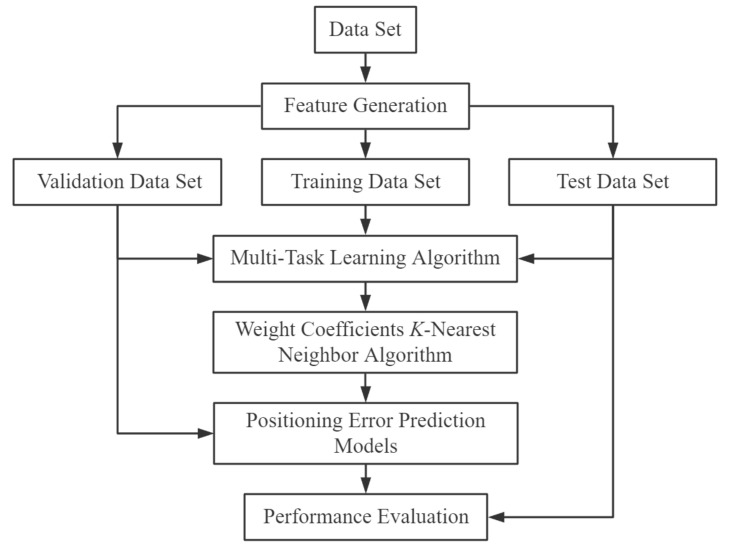
Positioning and localization method.

Researchers mainly use traditional machine learning algorithms or deep learning algorithms to realize classification and regression tasks. This paper combines a traditional machine learning [34,35,36] algorithm with a deep learning [37,38,39] algorithm. It proposes an adaptive multi-type fingerprints indoor positioning and localization method based on MTL and WCKNN to predict the position and location information of multi-type fingerprints. The stages of this method are shown in Figure 1. To begin with, the data set is pre-processed (standardized and normalized) to generate new features. The generated dataset is then randomly divided into 80% training data, 10% validation data, and 10% test data. In the next stage, training and validation data are presented to train the MTL model, and the relationship between the attributes of the training data is used to refine the parameters of the WCKNN algorithm. After the construction of MTL and WCKNN is completed, their performance is pre-evaluated using the validation data. Then, positioning error prediction models are established to forecast the confidence of the positioning results based on the results of the pre-evaluation. Finally, the performance of the proposed method is evaluated with test data according to the classification and regression performance metrics.

### 2.1. Miskolc IIS Hybrid IPS Dataset

**Figure 2:**
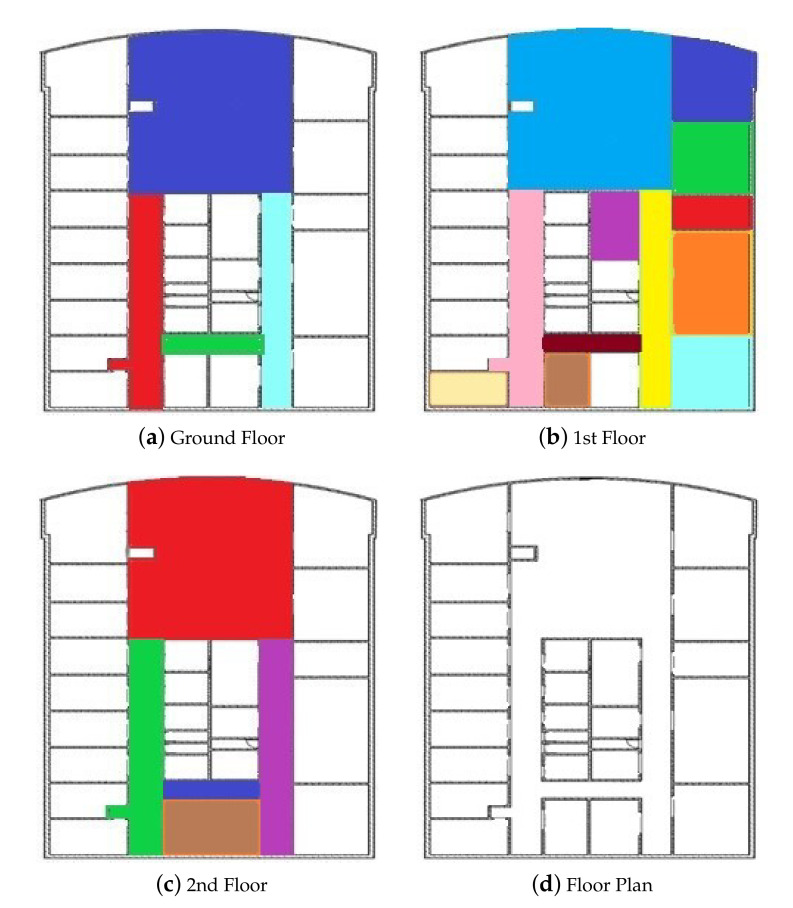
Structure of the Institute, where the colors distinguish each symbol location.

Miskolc IIS (Institute of Information Science) Hybrid IPS (Indoor Positioning System) data set [40,41] was recorded by the ILONA (Indoor Localization and Navigation) System [42] in the IIS Building at University of Miskolc. [43] The measurement environment is a grid-like layout of the three-story building, as seen in Figure 2. Figure 2a shows the ground floor, Figure 2b the first floor, Figure 2c the second floor, and Figure 2d shows the general floor plan of the building. The data set covers an area of 2106.16 m${}^{2}$, about 50% of the IIS building. The colors and lines of Figure 2 are used to separate areas. Some devices are deployed in the building, the Wi-Fi signal icons represent Wi-Fi access points, and the Bluetooth icons represent Bluetooth devices. The data set contains 1539 measurements, 32 Wi-Fi access points, 22 Bluetooth devices, and the magnetic field values [44].

In the dataset, each measurement point contains fingerprints with MF, Wi-Fi, and Bluetooth. Due to the loss of MF fingerprint data in some measurement points, it cannot be employed as an experimental sample. Moreover, the number of measurement points at some symbol locations is insufficient to construct a valid model. In decree to build a stable and reliable model, we remove measurement points with missing data and symbol locations with less than 30 measurement points. After processing, the dataset contains 1405 measurement points in 15 symbol locations, hence 15 classes can be distinguished. Table 1 shows the distribution of measurements in the building.

To facilitate data mining and processing, the processed dataset was exported to a comma-separated value (CSV) format. Table 2 shows the schema of the experimental data. The header holds the following fields: the symbol location, the absolute coordinates called *x*, *y*, *z*, the MF values, the Wi-Fi values, and the Bluetooth devices. The *x* is the vertical axis, and the *y* is the horizontal axis. Currently, the building is divided into a grid of 1 m × 1 m. The coordinates are determined based on the available grid. The measurement height of each floor is the same, and the measurement results are expressed by *z*, which are 1.5 m, 4.4 m, and 7.6 m, respectively. The MF gives back a three-dimensional real vector. The Wi-Fi values are placed between the 8–39 position where each position has a corresponding access point. In the dataset, the Wi-Fi value is either an integer in [−98,−20] or null, and null represents that the Wi-Fi access point was not observed at a given location. In order to boost data processing, −100 was chosen to represent unreachable access points. For each Bluetooth address, the corresponding binary (0,1) value denotes the visibility of the device [44].

Three different types of fingerprints are used in this paper to achieve localization and positioning. Wi-Fi and MF are deterministic fingerprints. In contrast, Bluetooth is a probabilistic fingerprint due to the relatively limited space coverage of Bluetooth hotspots. When a user with a Bluetooth-enabled device enters a specific range, the device recognizes the hotspot. In previous studies, it was found that the positioning and localization effects of multi-type fingerprints used by the model are better than a single type of fingerprint. Moreover, from the perspective of information theory, the more information we collect about a certain point, the more certain the symbol location and position coordinate of that point can be. Therefore, this article uses a data set named Miskolc IIS Hybrid IPS, and proposes an adaptive multi-type fingerprints indoor positioning and localization method based on MTL and WCKNN.

### 2.2. Multi-Task Learning

MTL is a learning algorithm that uses the useful information contained in multiple learning tasks to improve the performance of each task to a certain extent. The principal characteristics are as follows.

- Increase the generalization capabilities of the model. Different tasks have different expression patterns. A model that learns multiple tasks simultaneously can learn a more general representation, thereby improving the generalization capabilities of the model.
- Reduce the risk of getting stuck in local minima. In single-task learning, the backpropagation of gradients tends to become stuck in a local minimum. Nevertheless, the local minima of different tasks are in different positions in the MTL, and the hidden layer can escape from the local minima through the interaction between the tasks.
- Reduce over-fitting. MTL plays the same role as regularization by introducing an inductive bias, which can reduce the complexity of the model and the risk of over-fitting [27].

There are two mechanisms to realize MTL in neural networks: hard parameter sharing [45] and soft parameter sharing [46]. The hard parameter sharing is the most commonly used approach in neural network MTL. It is generally applied by sharing the hidden layers among all tasks while maintaining several task-specific output layers. For each task, there are features in the shared hidden layer that are relevant and irrelevant to the task, which can improve the generalization ability of the model. However, in soft parameter sharing, each task has its own model and parameters. The distance between the parameters of the model is then regularized to ensure the similarity of the model parameters. [27]

### 2.3. K-Nearest Neighbor

The KNN is a non-parametric and instance-based algorithm for classification and regression. The KNN algorithm searches the sample set for the *K* samples that are most similar to the object, thereby obtaining the results of classification and regression. The similarity is defined in terms of distance functions, such as Euclidean distance. In this paper, the Euclidean distance is used to calculate the similarity between samples. The *K* parameter is a positive integer that determines the number of the neighbors. The different selection of *K* value will directly affect the final classification and regression results.

The KNN classification algorithm is a classifier based upon a non-linear algorithm, and its output is a class membership. An object is assigned to the most common class among its *K* nearest neighbors by voting. If *K* is set to 1, the object is assigned directly to the class of the nearest neighbor, so the classification problem becomes a minimum search task. In the KNN regression algorithm, for a given object, its attribute value is the average of the attribute values of the *K* nearest neighbors.

## 3. Proposed Positioning and Localization Method

Under ideal conditions, MF fingerprint, Bluetooth fingerprint, and Wi-Fi fingerprint are used for positioning and localization simultaneously. The predicted location of the MF fingerprint, the predicted location of the Wi-Fi fingerprint, the predicted location of the Bluetooth fingerprint should be the same, as well as the predicted position of the MF fingerprint, the predicted position of the Wi-Fi fingerprint, and the predicted position of the Bluetooth fingerprint should also be similar. However, in reality, the results obtained may be substantially different due to various reasons (such as reflection, diffraction, diffuse scattering). Thus, in this paper, we propose an adaptive multi-type fingerprints indoor positioning and localization method based on MTL and WCKNN to realize the integration of MF, Wi-Fi, and Bluetooth fingerprints for positioning and localization.

**Figure 3:**
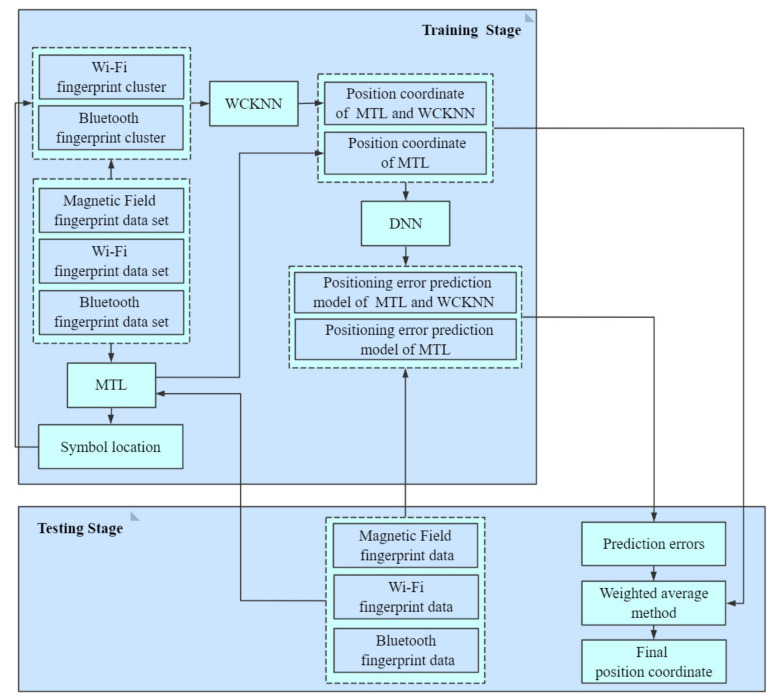
Flow diagram of adaptive multi-type fingerprints indoor positioning and localization method based on MTL and WCKNN.

The flow diagram of adaptive multi-type fingerprints indoor positioning and localization method based on MTL and WCKNN is shown in Figure 3. In the training stage, the MF, Wi-Fi, and Bluetooth fingerprint data were used to train an MTL model. The MTL model has two tasks, one is to predict the symbol location of multi-type fingerprints, and another is to predict the position coordinate of multi-type fingerprints. Subsequently, fingerprints with the same symbol location were collected from the database to construct a Wi-Fi fingerprint cluster and a Bluetooth fingerprint cluster, and another position coordinate was obtained by using WCKNN in the clusters. Finally, DNN-based positioning error prediction models are established based on the errors between each predicted position coordinate and the actual position coordinate. In the testing stage, the MF, Wi-Fi, and Bluetooth fingerprints of the test data were input into the MTL model to get symbol location and position coordinate. In the next stage, Wi-Fi and Bluetooth fingerprint clusters were established based on the symbol location obtained by MTL, and another position coordinate was obtained by using the WCKNN in the clusters. The fingerprint data is then input into the positioning error prediction models to determine the positioning errors of the two predicted position coordinates. Finally, the final position coordinate was obtained by fusing the two predicted position coordinates using a weighted average method whose weights were the positioning errors, as shown in Equation (1):

(1) $$Coordinate=\frac{\left(\frac{MPC}{MPC\_Error}+\frac{MWPC}{MWPC\_Error}\right)}{\left(\frac{1}{MPC\_Error}+\frac{1}{MWPC\_Error}\right)}$$

Here, *MPC* is the position coordinate predicted by the MTL, and *MWPC* is the position coordinate predicted by the MTL and WCKNN. *MPC_Error* is the positioning error of *MPC*, and *MWPC_Error* is the positioning error of *MWPC*. The predicted position coordinates are fused to realize adaptive positioning, which can improve positioning precision and stability.

### 3.1. Multi-Task Learning

The multi-type fingerprints of each measurement point potentially contain the symbol location and position coordinate information of the measurement point. A multi-type fingerprints indoor positioning and localization model based on MTL was designed in this paper, which can simultaneously predict the symbol location and position coordinate based on the provided multi-type fingerprints. Compared with single-task learning, MTL can additionally learn the potential relationship between two tasks, thereby improving the performance of the two tasks to a certain extent.

**Figure 4:**
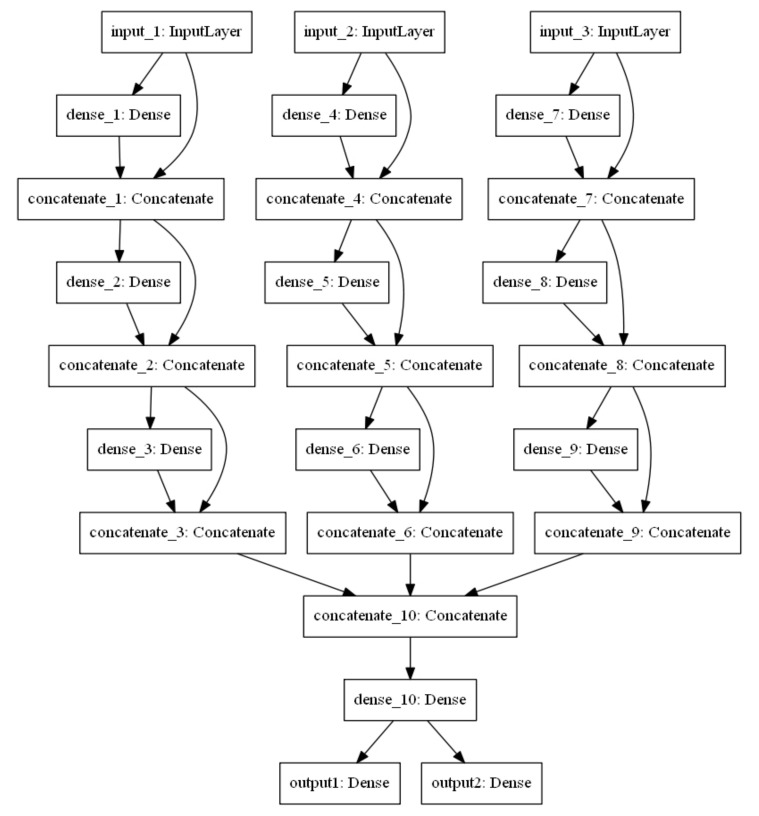
Architecture of MTL.

The architecture of the proposed MTL model is shown in Figure 4. The three input layers of the MTL model from left to right are input MF, Wi-Fi, and Bluetooth, respectively. The model has two output layers. The output layer on the left is a SoftMax layer that outputs the probability distribution of the current multi-type fingerprints belonging to the analyzed classes. The activation function of the output layer on the right is a linear function and outputs the possible position coordinate of the current multi-type fingerprints. Moreover, the input features of the “concentrate_1” to “concentrate_9” layers in the connection layers of the model are the fusion features of the input features and output features of the previous layer, which can avoid the loss of information during the propagation and reduce the risk of over-fitting the model. Furthermore, the MF, Wi-Fi, and Bluetooth fingerprint features are concatenated on the “concatenate_10” layer, which implements a hard parameter sharing mechanism and learns the potential relationship between them. The activation function of all hidden layers is the Rectified Linear Unit (ReLU), which can speed up network training, prevent gradient vanishing and explosion, and sparse network. Table 3 shows the main parameter settings of MTL, among which the loss function and loss weight of the two output layers are more worthy of our attention. The loss function of the output layer of the classification task is categorical_crossentropy, and the loss weight is 1. The loss function and loss weight of the output layer of the regression task were mean-square error (MSE) and 0.8, respectively. Therefore, the final loss is the weighted sum of the two losses, and the weighted by the loss weights. The proposed MTL model realizes the integration of MF, Wi-Fi, and Bluetooth fingerprints and applies the synergy between the tasks, which can improve the accuracy in predicting symbol locations and reduce errors in estimating position coordinates.

### 3.2. Weight Coefficients K-Nearest Neighbor

The details of the proposed WCKNN algorithm are explained in this section. The WCKNN is established based on the traditional KNN algorithm to adapt to the environment of multi-type signals.

(2) $$\begin{array}{cc} \; & S_{i}=\left[a_{i1},a_{i2},\cdots ,a_{in},b_{i1},\cdots ,b_{im},\cdots \right],\\ \; & S_{j}=\left[a_{j1},a_{j2},\cdots ,a_{jn},b_{j1},\cdots ,b_{jm},\cdots \right]. \end{array}$$

where $S_{i}$ is the vector of multi-type signals *i* and $S_{j}$ is the vector of multi-type signals *j*. The vector contains different types of signal features (such as *a, b*). The traditional KNN algorithm calculates the Euclidean distance between *i* and *j*, which is expressed as

(3) $$\begin{array}{c} D_{ij}=\sqrt{{\left(a_{i1}-a_{j1}\right)}^{2}+\cdots +{\left(a_{in}-a_{jn}\right)}^{2}+{\left(b_{i1}-b_{j1}\right)}^{2}+\cdots +{\left(b_{im}-b_{jm}\right)}^{2}+\cdots } \end{array}$$

Here, $D_{ij}$ is the distance between *i* and *j*. However, the traditional KNN algorithm does not consider the influence of different signal types on $D_{ij}$, which will inevitably inhibit the accurate selection of neighbors to a certain extent.

(4) $$\begin{array}{cc} D_{ij} & =\;\;w_{a}\sqrt{{\left(a_{i1}-a_{j1}\right)}^{2}+\cdots +{\left(a_{in}-a_{jn}\right)}^{2}}+w_{b}\sqrt{{\left(b_{i1}-b_{j1}\right)}^{2}+\cdots +{\left(b_{im}-b_{jm}\right)}^{2}}+\cdots \\ & =\;\;w_{a}D_{a_{i}a_{j}}+w_{b}D_{b_{i}b_{j}}+\cdots \end{array}$$

The WCKNN algorithm is proposed in this paper, as shown in Equation (4). By assigning weights to the distance between signals to express the influence of different types of signals on the similarity between multi-type signals, which helps to judge the similarity between multi-type signals. It is obvious that the size of the weight will determine the degree of influence.

#### 3.2.1. Weight Coefficients *K*-Nearest Neighbor Classification

Each measurement point is assigned its unique symbol location, but the same symbol location can include multiple measurement points. In order to study the relationship between multi-type fingerprints and symbol locations, the WCKNN classification algorithm is discussed in this section. According to Equation (4), we need to explore the relationship between fingerprints and symbol locations. For this, we use the Maximum Information Coefficient (MIC) to discover the functional relationship between the distance between fingerprints (Dis_Fingerprints) and the distance between symbol locations (Dis_Locations). The MIC is a statistical measure of the degree of association between variables. More precisely, define

(5) $$\begin{array}{c} MIC\left[X;Y\right]=\max_{x,y:xy\leq B}\frac{\sum_{x,y}p\left(x,y\right)\log_{}\frac{p(x,y)}{p(x)p(y)}}{\log_{}\left(\min\left(x,y\right)\right)} \end{array}$$

where *B* is dependent on the boundary between *X* and *Y*. It can be shown that the MIC lies in the range [0,1], where 0 represents no relationship between the variables, and 1 represents a noise-free relationship of any form, either linear or non-linear (such as regular graph modes).

**Figure 5:**
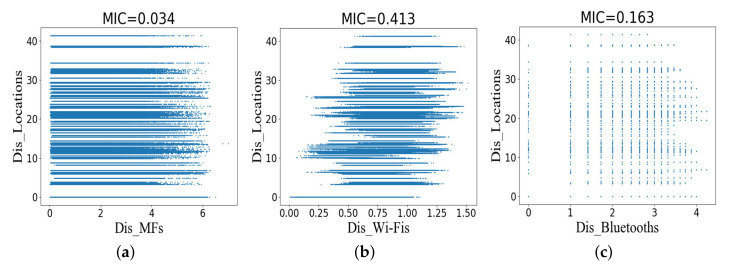
Scatter plots and MICs for the distance between fingerprints and the distance between symbol locations in the training dataset. (**a**) The distance between MFs and the distance between symbol locations. (**b**) The distance between Wi-Fis and the distance between symbol locations. (**c**) The distance between Bluetooths and the distance between symbol locations.

Figure 5 shows scatter plots and MICs for Dis_Fingerprints and Dis_Locations in the training dataset. The training dataset contains 1123 measurement points, producing a total of 631,126 sample pairs or scattered points. Since the symbol location is a category attribute and cannot perform mathematical operations, the difference between the center points of symbol locations is taken as Dis_Locations. As a result, a total of 106 Dis_Locations results were generated in 15 symbol locations. In the figure, we see scatter plots for particular pairs of variables, which we now discuss:

- Figure 5a has a low MIC. This is because they correspond to non-functional and one-to-many relationships between the distance between MFs (Dis_MFs) and Dis_Locations.
- The MIC of Figure 5b is 0.413, indicating that there is a certain relationship between the distance between Wi-Fis (Dis_Wi-Fis) and Dis_Locations.
- Figure 5c has an MIC of 0.163. The corresponding scatter plot makes it clear that there is a weak relationship between these two variables (the distance between Bluetooths or Dis_Bluetooths and Dis_Locations).

MIC reflects the relationship between fingerprints and symbol locations, not just linear. According to the analytical formula of Equation (4), we expect that there is a linear relationship between Dis_Fingerprints and Dis_Locations. The (Pearson) correlation coefficient is the simplest method to measure the linear correlation between *X* and *Y*. The correlation coefficient is defined as

(6) $$\begin{array}{c} corr\left[X,Y\right]=\frac{n\sum_{}x_{i}y_{i}-\sum_{}x_{i}\sum_{}y_{i}}{\sqrt{n\sum_{}{x_{i}}^{2}-{\left(\sum_{}x_{i}\right)}^{2}}\sqrt{n\sum_{}{y_{i}}^{2}-{\left(\sum_{}y_{i}\right)}^{2}}} \end{array}$$

where −1≤corr[X,Y]≤1. We expect that the correlation coefficient is related to the slope of the regression line, i.e., the coefficient *a* in the expression Y=aX+b. However, the fact that the correlation only reflects the noisiness and direction of a linear relationship, not the slope of that relationship [47].

**Figure 6:**
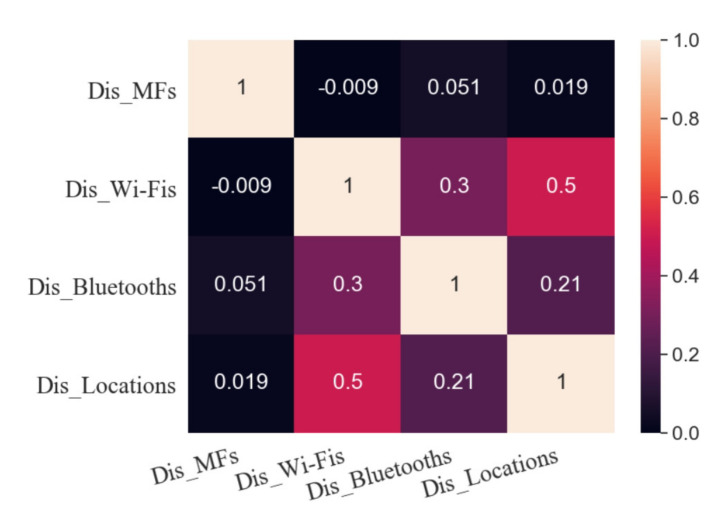
Pearson correlation coefficient between the distance between fingerprints and the distance between symbol locations.

Figure 6 shows the Pearson correlation coefficient between Dis_Fingerprints and Dis_Locations. The correlation coefficient between Dis_MFs and Dis_Locations is 0.019, and hence they are uncorrelated so that no linear function can be established between them. This result may be due to the influence of metal on walls and indoor equipment on MF distribution.

The correlation coefficient between Dis_Wi-Fis and Dis_Locations is 0.50, which indicates that they are moderately correlated. It is expressed as

(7) $$\begin{array}{c} D_{l_{i}l_{j}}=w_{1}D_{x_{i}x_{j}}+b_{1} \end{array}$$

Here, $D_{x_{i}x_{j}}$ is the distance between MF $x_{i}$ and MF $x_{j}$, and $D_{l_{i}l_{j}}$ is the distance between symbol location $l_{i}$ and symbol location $l_{j}$. In addition, *i* and *j* are multi-type fingerprints, including MF fingerprint, Wi-Fi fingerprint, and Bluetooth fingerprint. A weighting coefficient $w_{1}$ and a constant $b_{1}$ are provided to establish a linear functional relationship between Dis_Wi-Fis and Dis_Locations.

The correlation coefficient between Dis_Bluetooths and Dis_Locations is 0.21, and there is a weak correlation between them.

(8) $$\begin{array}{c} D_{l_{i}l_{j}}=w_{2}D_{y_{i}y_{j}}+b_{2} \end{array}$$

where *$y_{i}$* is the Bluetooth fingerprint *i* and *$y_{j}$* is the Bluetooth fingerprint *j*. Similarly, a linear functional relationship between Dis_Bluetooths and Dis_Locations by providing a weight coefficient $w_{2}$ and a constant $b_{2}$ is established.

(9) $$\begin{array}{c} D_{l_{i}l_{j}}=\frac{1}{2}\left(w_{1}D_{x_{i}x_{j}}+b_{1}+w_{2}D_{y_{i}y_{j}}+b_{2}\right) \end{array}$$

Equation (9) can be obtained by the addition operation of Equations (7) and (8). Since $b_{1}$ and $b_{2}$ are constants, their sum is also a constant, which does not affect the comparison of similarities between multi-type fingerprints. In order to reduce the amount of calculation, the sum is set to zero. Equation (9) can be simplified to Equation (10).

(10) $$\begin{array}{c} D_{l_{i}l_{j}}=w_{1}^{{}^{\prime }}D_{x_{i}x_{j}}+w_{2}^{{}^{\prime }}D_{y_{i}y_{j}} \end{array}$$

From the analyses described above, the correlation between symbol locations and Wi-Fi fingerprints is higher than that of MF fingerprints, and also higher than that of Bluetooth fingerprints. Also, Equation (10) shows the functional relationship among Dis_Wi-Fis, Dis_Bluetooths, and Dis_Locations; moreover, the weight coefficient represents the degree of correlation.

#### 3.2.2. Weight Coefficients *K*-Nearest Neighbor Regression

**Figure 7:**
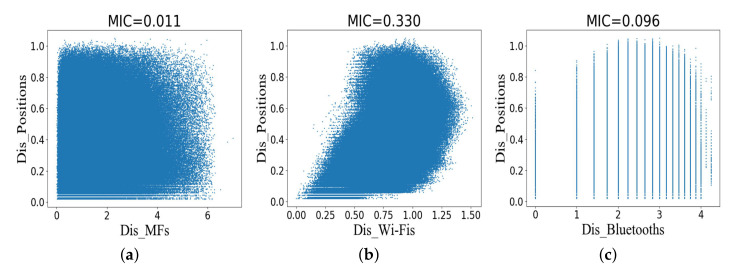
Scatter plots and MICs for the distance between fingerprints and the distance between position coordinates in the training dataset. (**a**) The distance between MFs and the distance between position coordinates. (**b**) The distance between Wi-Fis and the distance between position coordinates. (**c**) The distance between Bluetooths and the distance between position coordinates.

Each measurement point corresponds to an absolute position coordinate. In order to study the relationship between multi-type fingerprints and position coordinate, the WCKNN regression algorithm is discussed in this section. The distance between position coordinates (Dis_Positions) can be obtained using the Euclidean metric. Figure 7 shows scatter plots and MICs for Dis_Fingerprints and Dis_Positions in the training dataset. The degree of association between various types of fingerprints and position coordinates is weaker than that between symbol locations. Figure 7a shows that there is no relationship between Dis_MFs and Dis_Positions. Figure 7b,c show that the scatter maps have low regularity, especially Dis_Bluetooths and Dis_Positions.

**Figure 8:**
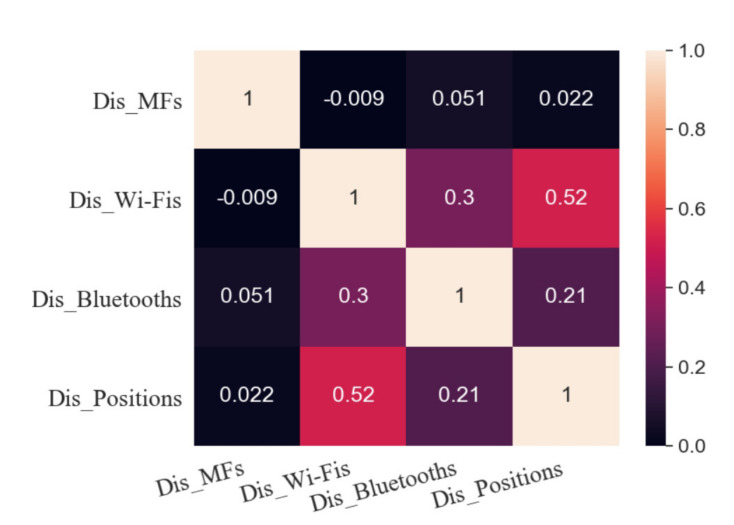
Pearson correlation coefficient between the distance between fingerprints and the distance between position coordinates.

To explore the linear relationship between various fingerprints and position coordinates, the Pearson correlation coefficient between Dis_Fingerprints and Dis_Positions is shown in Figure 8. Dis_Wi-Fis has the highest correlation with Dis_Positions, and its Pearson correlation coefficient is 0.52. The Pearson correlation coefficient between Dis_Bluetooths and Dis_Positions is 0.21, and there is a weak correlation between them. Similarly, there is no correlation between Dis_MFs and Dis_Positions.

(11) $$\begin{array}{c} D_{p_{i}p_{j}}=m_{1}D_{x_{i}x_{j}}+c_{1} \end{array}$$

(12) $$\begin{array}{c} D_{p_{i}p_{j}}=m_{2}D_{y_{i}y_{j}}+c_{2} \end{array}$$

(13) $$\begin{array}{c} D_{p_{i}p_{j}}=\frac{1}{2}\left(m_{1}D_{x_{i}x_{j}}+c_{1}+m_{2}D_{y_{i}y_{j}}+c_{2}\right) \end{array}$$

The derivation process of the WCKNN regression algorithm is similar to that of the WCKNN classification algorithm. Equations (11)–(13) are constructed. Where $p_{i}$ and $p_{j}$ are the position coordinates of multi-type fingerprints, and $D_{p_{i}p_{j}}$ is the distance between $p_{i}$ and $p_{j}$.

(14) $$\begin{array}{c} D_{p_{i}p_{j}}=m_{1}^{{}^{\prime }}D_{x_{i}x_{j}}+m_{2}^{{}^{\prime }}D_{y_{i}y_{j}} \end{array}$$

The functional relationship between Dis_Fingerprints and Dis_Positions is shown in Equation (14). The weight coefficient in the formula represents the degree of correlation between fingerprints and position coordinates. Moreover, the correlation between location coordinates and Wi-Fi fingerprints is higher than that of MF fingerprints, and also higher than that of Bluetooth fingerprints.

The analytical expression of Equation (4) is the same as those of Equations (10) and (14). Therefore, the proposed WCKNN algorithm is suitable for the environment of multi-type fingerprints in this paper. We can obtain the values of $D_{x_{i}x_{j}}$, $D_{y_{i}y_{j}}$, $D_{l_{i}l_{j}}$, and $D_{p_{i}p_{j}}$ from the training data, so that the optimal solution of the weight coefficients of Equations (10) and (14) can be obtained by the backpropagation neural network.

### 3.3. Positioning Error Prediction

**Figure 9:**
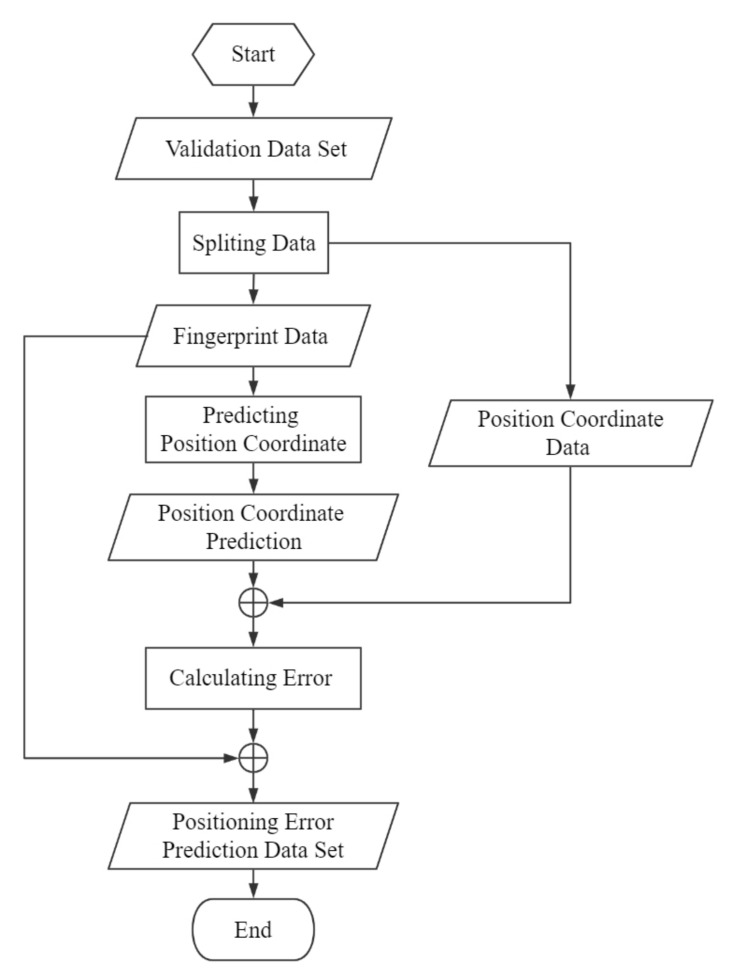
Flow diagram of the process of constructing the positioning error prediction dataset.

To prevent empirical errors, we use validation data instead of training data to construct training datasets for Positioning error prediction models. As shown in Figure 9, to start with, the validation data are split into two parts: fingerprint data and position coordinate data. In the next stage, the fingerprint data are presented to the proposed algorithms to get position coordinates *MPC* and *MWPC*. Then, the error between each predicted position coordinate and the actual position coordinate is calculated using the Euclidean metric, and then *MPC_Error* and *MWPC_Error* are obtained, respectively. Finally, combining each positioning error with fingerprint data to construct the positioning error prediction datasets.

**Figure 10:**
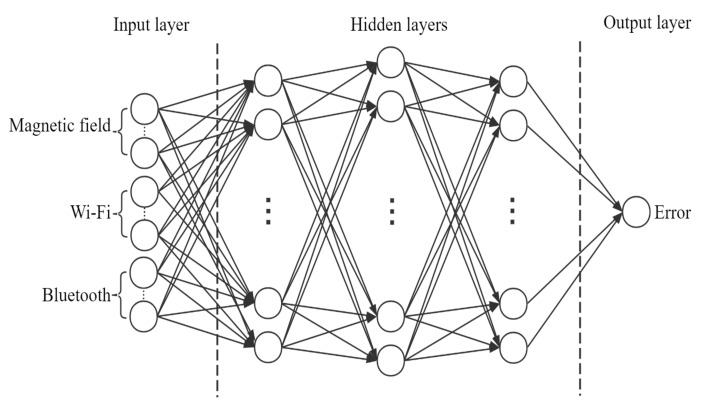
Architecture of DNN.

The DNN is selected to build positioning error prediction models. Figure 10 represents the architecture of the positioning error prediction model for forecasting *MPC_Error*. The sample of the positioning error prediction dataset is not too large; therefore, a simple five-tier DNN architecture is used, which consists of three hidden layers to do the regression. The model inputs MF, Wi-Fi, and Bluetooth fingerprints in the input layer, and its number of neurons is 60. Then, three hidden layers are designed to be 128 neurons, 256 neurons, and 128 neurons, respectively. The number of neurons in the output layer of the model is equal to one, and the prediction error is output. The architecture of the positioning error prediction model for forecasting *MWPC_Error* is similar to that for forecasting *MPC_Error*. As described in Section 3.2.2, the data used by the WCKNN regression algorithm only involves Wi-Fi and Bluetooth fingerprints. Therefore, for the positioning error prediction model used to predict *MPC_Error*, only Wi-Fi and Bluetooth fingerprints are input in the input layer, the connection between the MF and the neurons is cut off in the hidden layer, while the output layer remains unchanged.

## 4. Results and Discussion

In this section, the positioning and localization results based on the proposed method were presented. The data set was split (80% training,10% validation, 10% test), 1123 samples were available for training, 141 for validation, and 141 for testing. The main factor in model classification performance evaluation is the accuracy based on the test data. Accuracy refers to the degree of consistency between predicted results and measured results, which provides us with more certainty whether an unknown measurement point can be correctly classified.

(15) $$\begin{array}{c} Accuracy\left(c\right)=\frac{|c_{Correct}|}{|c_{Correct}\left|+\right|c_{Incorrect}|} \end{array}$$

The formula in Equation (15) is the accuracy of a given c class. $c_{Correct}$ indicates that actual C is predicted as C, and $c_{Incorrect}$ indicates that actual C is predicted as non-C. In order to determine the accuracy of the proposed method, the proportion of all samples correctly predicted needs to be calculated.

To evaluate the regression performance of the model, we employed mean average error (MAE), maximum error (ME), and root-mean-square error (RMSE), which are common evaluation indicators used in regression models. As shown in Table 4, it represents the sample standard deviation of the differences between predicted position coordinate (${\hat{y}}_{i}$) and measured position coordinate ($y_{i}$).

### 4.1. Effect of Using Multi-Task Learning Alone

To study the prediction performance of the proposed model MTL, in this section, MTL was used to achieve multi-type fingerprints indoor positioning and localization. This way was named the multi-type fingerprints indoor positioning and localization method based on MTL in this work. The principle is to use the useful information contained in the tasks of predicting symbol locations and position coordinates to improve their performance to a certain extent.

**Figure 11:**
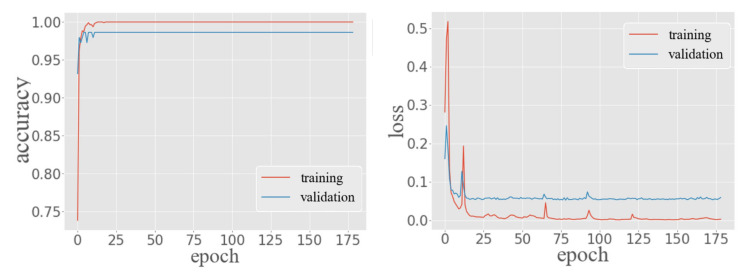
Performance of MTL in training and validation data. Red curves indicate training data, and blue curves indicate validation data. (**Left**): Classification task. (**Right**): Regression task.

Figure 11 shows the performance of MTL in training and validation data. The classification task of the MTL model produced 100% accuracy in the training data and 98.60% accuracy in validation data. In the regression task, the training loss of the MTL had steadily decreased around 0 after 175 epochs. In contrast, the validation loss dropped around 0.55. Moreover, it can be seen from the figure that training loss and validation loss have been declining, and validation loss has not picked up. This indicates that the architecture and parameter settings of the proposed MTL model are successful.

Since follow-up experiments need to use MTL, this paper chooses the model with more frequent test results to represent the performance of MTL. The MTL provided 98.58% accuracy of predicting the symbol locations of multi-type fingerprints in the test data. In terms of predicting the position coordinates, the MAE of the MTL was 2.46 m with a ME of 11.45 m and an RMSE of 3.71 m. At the same time, the accuracy of the test data is very close to that of the training data and validation data, which further proves that the model has no over-fitting and the architecture and parameter setting are effective.

### 4.2. Effect of K-Nearest Neighbor Based on Different Types of Fingerprints

From the perspective of information theory, the more information we know about a certain point, the more certain the symbol location and position coordinate of that point can be. However, if we collect noise, it is not only useless for our prediction but also affects our correct judgment. According to the analysis in Section 3.2, we know that the MF fingerprint is a kind of noise to the KNN algorithm. Therefore, in this section, we discuss the impact of Wi-Fi, Bluetooth, and multi-type fingerprints on the performance of the KNN algorithm.

**Figure 12:**
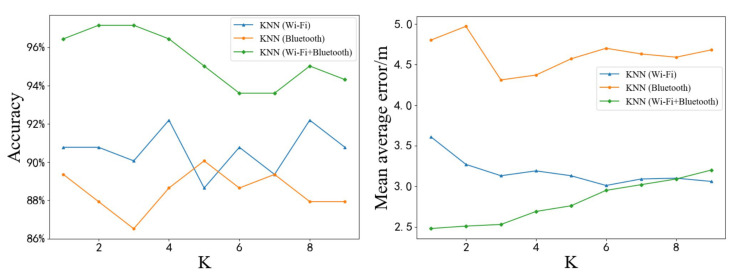
Test results of KNN based on different types of fingerprints at different *K* values. Blue curves represent the KNN algorithm based on Wi-Fi fingerprint, orange curves represent the KNN algorithm based on Bluetooth fingerprint, and green curves represent the KNN algorithm based on Wi-Fi and Bluetooth fingerprints. (**Left**): Accuracy of predicted symbol location. (**Right**): MAE of predicted position coordinate.

To determine the efficiency of the algorithm, the value of *K* in the KNN algorithm was changed to make the algorithm more efficient in the IIS environment. The value of *K* was set from 1 to 9. Figure 12 shows the test results of the KNN algorithm based on different types of fingerprints at different *K* values. The left figure in Figure 12 shows the accuracy of predicting symbol locations. When *K*= 4 and *K*= 8, the highest accuracy of the KNN algorithm based on Wi-Fi fingerprint was 92.20%, which is an increase of 1.93% compared with the highest accuracy of the KNN algorithm based on Bluetooth fingerprint. In contrast, the highest accuracy of the KNN algorithm based on Wi-Fi and Bluetooth fingerprints was 97.16%. From the right figure in Figure 12, it can be seen that the lowest MAE of the KNN algorithm based on Wi-Fi fingerprint was 3.01 m at *K* = 6, which is a decrease of 1.20 m compared to the lowest MAE of the KNN algorithm based on Bluetooth fingerprint. In contrast, the lowest MAE of the KNN algorithm based on Wi-Fi and Bluetooth fingerprints was 2.5 m at *K* = 1. However, as the value of *K* increases, the MAE of the KNN algorithm based on Wi-Fi and Bluetooth fingerprints gradually increases, while the MAE of the KNN algorithm based on Wi-Fi fingerprint has been declining. When *K* was greater than 8, the precision of the KNN algorithm based on Wi-Fi fingerprint exceeded the precision of the KNN algorithm based on Wi-Fi and Bluetooth fingerprints. This is not only due to the influence of *K* value, but also more likely due to the increase of *K* value, resulting in Bluetooth fingerprint gradually become the noise of KNN algorithm based on Wi-Fi and Bluetooth fingerprints, rather than useful information.

In summary, when Wi-Fi and Bluetooth fingerprints are used together, the positioning effect is better than that when used alone. In theory, increasing the complexity of information can enhance the performance of the algorithm. However, it is still necessary to find an effective method to process this information, otherwise, the increase in information complexity may bring more uncertainty to the algorithm.

### 4.3. Effect of Using Weight Coefficients K-Nearest Neighbo Alone

This section analyzes the effect of the multi-type fingerprints indoor positioning and localization method based on WCKNN. Its principle is to consider the influence of different types of fingerprints on the similarity between multi-type fingerprints, so as to judge the similarity between multi-type fingerprints accurately.

The weight coefficients of Equations (10) and (14) can be obtained by the backpropagation neural network. In order to reduce the computational complexity, the optimal solution of the weight coefficients after normalization is that $w_{1}^{{}^{\prime }}$ is 0.718, $w_{2}^{{}^{\prime }}$ is 0.282, $m_{1}^{{}^{\prime }}$ is 0.897, and $m_{2}^{{}^{\prime }}$ is 0.103. Where $w_{1}^{{}^{\prime }}$ is greater than $w_{2}^{{}^{\prime }}$, it indicates that the similarity between symbol locations depends more on the similarity between Wi-Fi fingerprints. Therefore, the correlation between symbol locations and Wi-Fi fingerprints is stronger than that with Bluetooth fingerprints. Similarly, it can be seen from the comparison between $m_{1}^{{}^{\prime }}$ and $m_{2}^{{}^{\prime }}$ that the similarity between position coordinates depends more on the similarity between Wi-Fi fingerprints, and the correlation between position coordinates and Wi-Fi fingerprints is stronger than that with Bluetooth fingerprints. These conclusions are the same as those obtained from the MIC and the correlation coefficient in Section 3.2, so the obtained weight coefficients are credible. The theoretical analysis proves that compared with the KNN algorithm, the proposed WCKNN algorithm considers the impact of different types of fingerprints on the prediction target when determining the similarity between multi-type fingerprints. In order to further validate the analytical results and performance, the WCKNN is compared with the KNN algorithm from two aspects: the accuracy of predicting symbol locations and the MAE of predicting position coordinates.

**Figure 13:**
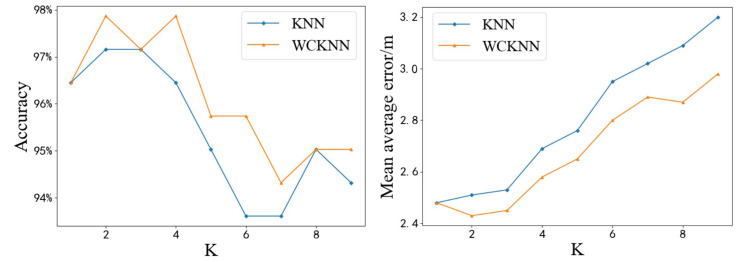
Test results of KNN and WCKNN at different *K* values. Orange curves indicate the KNN algorithm, and blue curves indicate the WCKNN algorithm. Left: Accuracy of predicted symbol location. Right: MAE of predicted position coordinate.

Figure 13 shows the test results of KNN and WCKNN at different *K* values. It can be seen that WCKNN has a better prediction effect. Its prediction accuracy and precision are significantly better than KNN. The left figure in Figure 13 shows the accuracy of predicting symbol locations. When *K* = 2 and *K* = 4, the highest accuracy of WCKNN was 97.87%, which is an increase of 0.71% compared with the highest accuracy of KNN. Moreover, when *K* = 6, the maximum difference in accuracy between the two algorithms is 2.13%. The WCKNN classification algorithm had higher accuracy in predicting symbol locations than the KNN classification algorithm. From the right figure in Figure 13, it can be seen that the lowest MAE of WCKNN was 2.43 m at *K* = 2, which is a decrease of 0.05 m compared to the lowest MAE of KNN. Moreover, when *K* = 9, the maximum difference in MAE between the two algorithms was 0.22 m. The WCKNN regression algorithm had lower MAE in predicting position coordinates than the KNN regression algorithm. The test data verified that the performance of the WCKNN algorithm is better than the MTL algorithm.

### 4.4. Multi-Type Fingerprints Indoor Positioning and Localization Method Based on MTL and WCKNN

By comparing the test results of MTL and WCKNN, it was found that MTL achieves higher accuracy in predicting symbol locations, but WCKNN has better performance in predicting position coordinates than MTL. The clusters are constructed based on the symbol location obtained by MTL to reduce the positioning range of the WCKNN algorithm.

**Figure 14:**
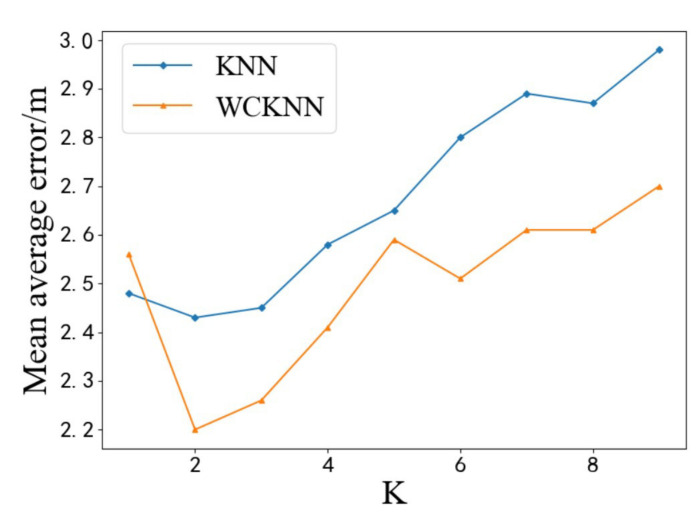
Test results of MTL_WCKNN and WCKNN in predicting position coordinates at different *K* values. The blue curve indicates the WCKNN algorithm, and the orange curve indicates the MTL_WCKNN algorithm.

This section analyzes the multi-type fingerprints indoor positioning and localization method based on MTL and WCKNN (MTL_WCKNN). Figure 14 shows the test results of MTL_WCKNN and WCKNN in predicting position coordinates at different *K* values. When *K* = 2, both MTL_WCKNN and WCKNN had the lowest MAEs of 2.11 m and 2.43 m, respectively, and the difference between them is 0.32 m. Moreover, when *K* = 6 and *K* = 9, the maximum difference in MAE between the two algorithms is 0.36 m. Compared with MTL and WCKNN, the MTL_WCKNN had a better positioning performance. We can see from the above experimental results that when the value of *K* is 2, both WCKNN and MTL_WCKNN algorithms have the best positioning performance. In order to obtain more reliable study results, in the following experiments, the value of *K* of the WCKNN and MTL_WCKNN algorithms was set to 2.

**Figure 15:**
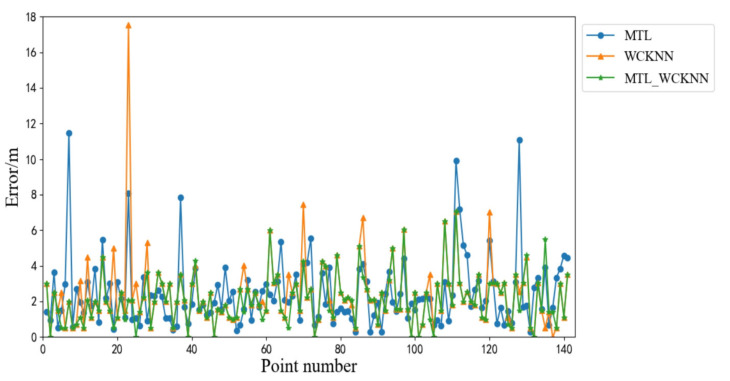
Positioning errors of MTL_WCKNN, MTL, and WCKNN.

Figure 15 shows the positioning errors of MTL_WCKNN, MTL, and WCKNN. The positioning effect of MTL_WCKNN is more accurate than those of MTL and WCKNN. Its number of large errors is less than those of MTL and WCKNN. The MEs of MTL and WCKNN were 11.45 m and 17.52 m, respectively, while the ME of MTL_WCKNN was 7.07 m, indicating that MTL_WCKNN has better positioning performance and can prevent larger errors. Moreover, the MAEs of MTL, WCKNN, and MTL_WCKNN were 2.46 m, 2.44 m, and 2.11 m, respectively. Compared with MTL and WCKNN, the MTL_WCKNN increased the positioning precision by 0.35 m and 0.33 m, respectively. The RMSEs of MTL, WCKNN, and MTL_WCKNN were 3.71 m, 4.06 m, and 1.97 m, respectively. It can be seen that the positioning effect of MTL_WCKNN was improved to a certain extent when compared with that of MTL and WCKNN.

**Figure 16:**
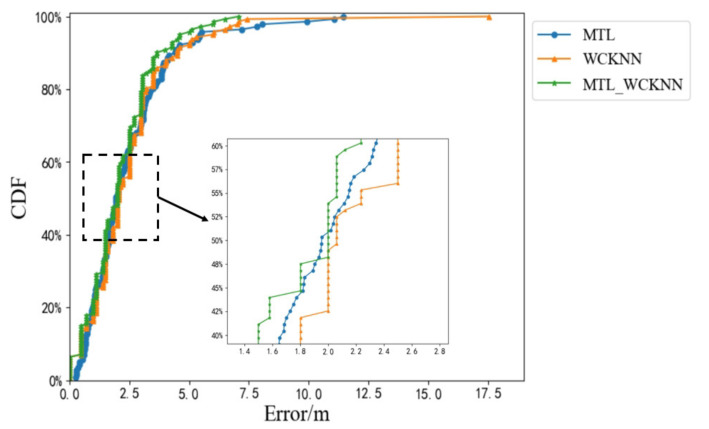
CDFs of positioning errors of MTL_WCKNN, MTL, and WCKNN.

Figure 16 shows the cumulative distribution functions (CDFs) of the positioning errors of MTL_WCKNN, MTL, and WCKNN. CDF is the integral of the probability density function, which can completely describe the probability distribution of the average error. When MTL and WCKNN were used together, its positioning effect was better than that when used independently. The ME of MTL_WCKNN was smaller than that of MTL and WCKNN, as well as there are fewer large errors. Therefore, the positioning effect of MTL_WCKNN was better than that of MTL and WCKNN.

### 4.5. Adaptive Multi-Type Fingerprints Indoor Positioning and Localization Method Based on MTL and WCKNN

The positioning results of MTL_WCKNN and WCKNN are limited by the surrounding neighbors, which leads to small discontinuities in their positioning error intervals. As shown in Figure 16, the CDF curves of MTL_WCKNN and WCKNN show a type of stairs to rise. In contrast, the MTL is not affected by the surrounding samples, and its CDF curve shows a smooth type to rise. In order to mitigate the impact of the surrounding neighbors on the object estimation range, the positioning error prediction models are used to provide the positioning errors as the confidence of position coordinates. The position coordinates obtained by MTL and MTL_WCKNN are then fused by the weighted average method whose weights are the positioning errors, thereby achieving adaptive positioning.

**Figure 17:**
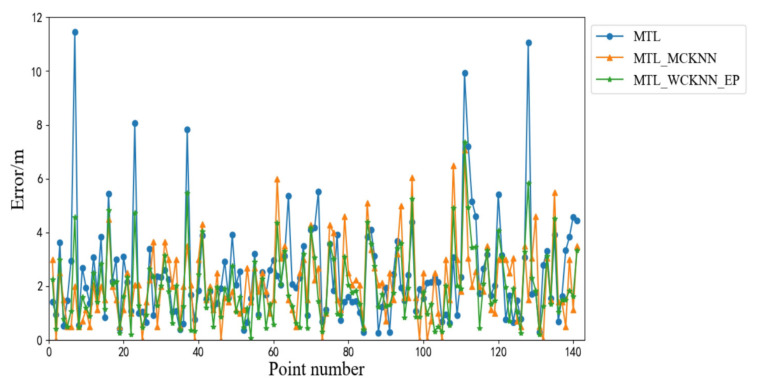
Positioning errors of MTL_WCKNN_EP, MTL, and MTL_WCKNN.

This section analyzes the adaptive multi-type fingerprints indoor positioning and localization method based on MTL and WCKNN (MTL_WCKNN_EP). Figure 17 shows the positioning errors of MTL_WCKNN_EP, MTL, and WCKNN. The MTL_WCKNN_EP was achieved with an MAE of 1.95 m, an ME of 8.36 m, and an RMSE of 1.92 m. The positioning effect of MTL_WCKNN_EP was the best among MTL, MTL_WCKNN, and MTL_WCKNN_EP. The positioning precision and stability were better than those of the MTL and MTL_WCKNN.

**Figure 18:**
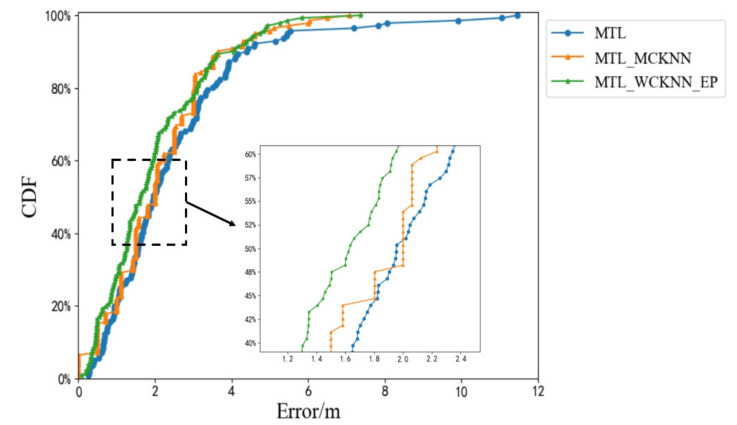
CDFs of positioning errors of MTL_WCKNN_EP, MTL, and MTL_WCKNN.

The CDFs of the positioning errors of MTL_WCKNN_EP, MTL, and MTL_WCKNN are shown in Figure 18. It can be seen that MTL_WCKNN_EP has the best positioning effect. With the help of the positioning error prediction models, the CDF curve of MTL_WCKNN_EP showed a smooth type to rise, which mitigated the influence of the surrounding samples to a certain extent and improved the positioning precision. The positioning effect of MTL_WCKNN_EP was superior to MTL_WCKNN. The ME of MTL_WCKNN_EP was 8.36 m, which was a decrease of 3.09 m when compared with MTL. However, the ME of MTL_WCKNN was 7.07 m, which is smaller than that of MTL_WCKNN_EP. Although the ME of MTL_WCKNN is better than that of MTL_WCKNN_EP, we can still see from the figure that MTL_WCKNN_EP can better avoid the occurrence of larger errors.

Table 5 shows the statistics for the positioning errors of MTL, WCKNN, MTL_WCKNN, and MTL_WCKNN_EP. The MAE of MTL_WCKNN was 2.11 m with an RMSE of 1.97 m. It was found that although MTL_WCKNN showed a more significant improvement compared to MTL and WCKNN, this improvement was limited to a certain extent by the distribution of samples. The MAE and RMSE of MTL_WCKNN_EP were 1.95 m and 1.92 m, respectively. Compared with MTL and WCKNN, the positioning precision increased by 20.73% and 20.08%, and the RMSE decreased by 1.79 m and 2.14 m, respectively. Its positioning precision and stability were significantly improved when compared with MTL and WCKNN. Compared with MTL_WCKNN, the positioning precision of MTL_WCKNN_EP increased by 7.58%, and the RMSE decreased by 0.05 m. The positioning effect of MTL_WCKNN_EP also showed a definite improvement when compared with MTL_WCKNN.

There is a certain relationship between model performance and model complexity. Table 6 shows statistics about the complexity of MTL, WCKNN, MTL_WCKNN, and MTL_WCKNN_EP. The parameters of the WCKNN classification algorithm and the WCKNN regression algorithm only involve the value of *K* and two-weight coefficients. In contrast, the MTL method is based on a neural network and has a huge amount of parameters, and the parameter quantity is proportional to the depth and width of the neural network, as well as the input feature quantity. MTL_WCKNN is formed by the series connection of MTL and WCKNN, as shown in the table, its parameter is equal to the sum of MTL and WCKNN. MTL_WCKNN_EP has the largest number of parameters, because it adds a neural network-based DNN model to achieve adaptive positioning. As the parameter and complexity of the model increase, the positioning performance is also significantly improved. Moreover, although neural network-based methods have higher complexity than traditional machine learning methods, they also bring higher accuracy and stability to positioning.

### 4.6. Comparison of Findings with Other Studies

The present study of the Miskolc IIS Hybrid IPS dataset focused more on the classification of symbol location. The most successful method based on deep learning algorithms seems to be the Artificial Neural Network (ANN) proposed by Tamas et al. [43]. In [43], the learning rate, momentum and other parameters of ANN algorithm are selected differently, and the optimal result is 96.77%. However, the accuracy of the proposed MTL algorithm in this paper is 98.58%, which is 1.81% higher than that of the ANN. Based on the traditional machine learning algorithm, the four methods of KNN, Naive Bayes, Decision Tree, and Rule Induction were experimented and compared in [44]. The KNN classifier had the best performance (92.26%) when the *K* parameter was 3. However, through data pre-processing, the highest accuracy of the WCKNN algorithm proposed in this paper is 97.87%, which is 5.61% higher than the KNN. According to the findings, it is obvious that our proposed algorithms can achieve higher performance than the existing algorithms.

## 5. Conclusions

This paper studies a combination algorithm of a traditional machine learning algorithm and a deep learning algorithm for positioning and localization, and proposes an adaptive multi-type fingerprints indoor positioning and localization method based on MTL and WCKNN to realize the integration of MF, Wi-Fi, and Bluetooth fingerprints. Moreover, the method realizes adaptive positioning by judging the confidence of the positioning results. Through our experiments, this fusion method shows a better positioning effect than MTL or WCKNN algorithm alone. Its positioning precision and stability were also higher than that of a multi-type fingerprints indoor positioning and localization method based on MTL and WCKNN. In addition, it is clear that the proposed algorithms have a better performance than other algorithms, including the ANN algorithm and the KNN algorithm. The proposed method can make full use of the MF, Bluetooth, and Wi-Fi in indoor environments to realize indoor positioning and localization with higher accuracy, precision, and stability. Therefore, this method has the potential to be an effective and general method for the future design of high-performance positioning and positioning services. However, the amount of computation and storage required is larger than that required for ordinary indoor positioning and localization method. Thus, future research is needed to investigate how to optimize our algorithms and establish sparse neural networks.

## Figures and Tables

**Table 1:** Number of measured points in symbol locations.

Symbol Location	# of Number	Symbol Location	# of Number
Ground Floor West Corridor	68	Ground Floor Lobby	208
Ground Floor East Corridor	103	1st Floor Lobby	151
2nd Floor West Corridor	86	2nd Floor Lobby	175
2nd Floor East Corridor	86	Lecture Hall XXVI	77
1st Floor West Corridor	56	Lab101	70
1st Floor East Corridor	59	Lab103	108
Lecture Hall 205	63	Lab104	63
Lab 115	32		

**Table 2:** The schema of the experimental data.

Location Information	Position Information	Measurements		
**Symbol Location**	**Absolute Coordinates**	**Magnetic Field**	**Wi-Fi**	**Bluetooth**
1	2–4	5–7	8–39	40–62

**Table 3:** Main parameters of MTL.

Learning Rate	Batch Size	Optimizer	Loss Function	Loss Weight
0.001	65	Adam	Categorical_crossentropy	1
			Mean-square error (MSE)	0.8

**Table 4:** Regression metrics.

Metric	Equation
Mean Average Error (MAE)	MAE=$\frac{1}{n}\sum_{i=1}^{n}|y_{i}-{\hat{y}}_{i}|$
Maximum Error (ME)	ME=$\max_{1\leq i\leq n}|y_{i}-{\hat{y}}_{i}|$
Root-Mean-Square Error (RMSE)	RMSE=$\sqrt{\frac{\sum_{i=1}^{n}{\left(y_{i}-MAE\right)}^{2}}{n}}$

**Table 5:** Statistical results of positioning errors (m).

Method	MAE	ME	RMSE
MTL	2.46	11.45	3.71
WCKNN	2.44	17.52	4.06
MTL_WCKNN	2.11	7.07	1.97
MTL_WCKNN_EP	1.95	8.36	1.92

**Table 6:** Statistical results of complexity.

Method	MTL	WCKNN	MTL_WCKNN	MTL_WCKNN_EP
Parameters	1,223,918	3	1,223,921	3,143,251
